# Pollen gene flow, male reproductive success, and genetic correlations among offspring in a northern red oak (*Quercus rubra* L.) seed orchard

**DOI:** 10.1371/journal.pone.0171598

**Published:** 2017-02-06

**Authors:** Lisa Alexander, Keith Woeste

**Affiliations:** 1 Department of Forestry and Natural Resources, Purdue University, West Lafayette, Indiana, United States of America; 2 USDA Forest Service Northern Research Station, Hardwood Tree Improvement and Regeneration Center, Purdue University, West Lafayette, Indiana, United States of America; Austrian Federal Research Centre for Forests BFW, AUSTRIA

## Abstract

Northern red oak is a high-value hardwood used for lumber, furniture and veneer. Intensively managed northern red oak seed orchards are required to obtain genetic gain for trait improvement. Data from conifer seed orchards and natural and managed stands of hardwood trees have shed light on the distance over which pollen can move, and underscore the need for managerial attention to seed orchard design, placement, and maintenance. We used eleven microsatellite markers to investigate pollen gene flow, female mate choice, and male reproductive success in a clonal seed orchard of northern red oak based on paternity analysis of seed orchard offspring in progeny tests. Nearly all (93%) offspring were sired by a male parent within the seed orchard. The mean number of male parents per year was 69.5, or 47.6% of all clones in the seed orchard. Female clones in the early phenology group had more offspring sired from extra-orchard pollen (13%) than clones in the intermediate (5%) and late (1%) phenology groups. Distance was the largest influence on pollination success, and pollination occurred most often by male trees in the same subline as the maternal tree. Males in the early phenology group sired more offspring overall in the progeny pool and more offspring per mother tree than males in the intermediate or late phenology groups. Average genetic correlations among all OP progeny ranged between 0.2557 and 0.3529 with a mean of 0.28±0.01. The importance of progeny test genotyping for northern red oak improvement likely is increasing with the demand for improved varieties. The current study demonstrated the feasibility of *post hoc* assembly of full-sib families for genetic analysis.

## Introduction

Three major findings can be distilled from research into mating patterns in conifer seed orchards. First, data from both seed orchards and natural stands show that pollen gene flow is substantial and that rates of self-fertilization are low (*Pinus taeda* [1]; *Pinus attenuata* [2]; *Pseudotsuga menziezii* [3–5]). Second, maximum-likelihood models of paternity analysis indicate that distance and phenology are the best predictors of mating frequency (*Pseudotsuga menziesii* [4,6]; *Pinus attenuata* [2]). Third, mating success can vary greatly among clones (*Pinus contorta* [7]; *Pseudotsuga menziezii* [4,5]). Although the effective number of males per females in both seed orchards and natural stands of conifers is generally greater than 10 [2,4,8], Slavov *et al*. [5] noted that a high effective number of males can result from a high number of clones in an orchard rather than even paternal contribution.

Studies of pollination often report a high percentage of offspring with male parents outside the study sites. Dow and Ashley [9] observed 57% of progeny were sired by trees at least 100 m from a twenty-year old natural stand of bur oak (*Quercus macrocarpa*). Furthermore, they found that a tree’s location in the stand (on the edge or in the middle) had no effect on the rate of pollen contamination. Buiteveld *et al*. [10] found that the rate of pollen contamination was 70% in a seed orchard that was 400m away from the nearest oak tree. Streiff *et al*. [11] observed that 65% of *Quercus robur* offspring and 69% of *Q*. *petraea* offspring were the result of pollinations from outside a 26 year-old natural mixed stand. Working in a natural stand of *Juglans nigra*, Robichaud [12] found that nearly 75% of pollinations were from males outside of the study site and probably greater than 1 km away. These results shed some light on the distance over which pollen can move, and underscore the need for managerial attention to seed orchard placement and maintenance.

### Pollination success

Unequal contributions of seed orchard clones to seed crops and departures from random mating are two major ways in which the genetic efficiency of seed orchards can be adversely affected [13]. Specifically, differential mating success of individual males results in a reduced effective population size (*N*_*e*_) of male parents compared to the census population [4]. Dow and Ashley [14] examined three maternal bur oaks in a twenty-year old natural stand and found that the number of acorns pollinated by near neighbors (0–50 m away) did not vary among trees, and that pollen donors were randomly distributed throughout the stand, with the exception of one maternal tree that was very close to its neighbors. These results showed that number of pollinations is not a linear function of proximity, but that a strong near-neighbor bias exists up to 50 m in distance [15,16]. Within the bur oak stand, 38 of 62 trees sired acorns, and self-pollination was virtually non-existent. More or fewer neighboring trees, genetic relatedness, cardinal direction from seed parent, and DBH of pollen donor had no influence on pollination success [9,14]. Working in a *Quercus robur* clonal seed orchard, Buiteveld *et al*. [10] found that the number of pollinations by clones roughly corresponded to the number of ramets of the clones in the orchard. Although only 19 of 56 potential pollen donors contributed to pollinations, there was no differential reproductive success among the contributing pollen donors, and no difference in pollen contamination between low, middle, and high crown levels or between cardinal directions of the crown.

### Genetic correlations among offspring

Selection based on open-pollinated (OP) family progeny testing is predominant in tree breeding–especially in the beginning stages of a breeding program aimed at backwards selection for seed orchard establishment or at forward selection for selecting second-generation plus trees [17]. The rate of genetic gain by selection depends in part by obtaining precise and unbiased estimates of genetic variances [18], which are based on an average genetic correlation of 0.25 for the presumably half-sibling offspring in each OP family. Seeds harvested from a single female parent are only true half-sibs if none resulted from selfing, and if all were sired by different males. If these conditions are not met, the average genetic correlation will be higher than 0.25, and estimates of additive genetic variance, heritabilities, and genetic gains will be biased [19].

### Paternity analysis

In studies of parentage in oak, between 4 and 6 unlinked microsatellite loci have been used to obtain exclusion probabilities as high as 99% [9,10,11,20]. The goal of the current study was to use a suite of microsatellite markers previously tested in northern red oak to investigate pollen gene flow, female mate choice, and male reproductive success in a clonal seed orchard of northern red oak based on paternity analysis of seed orchard offspring in progeny tests. The specific objectives of the study were to determine i) the percentage of clones acting as pollen parents each year and the influence of male phenology, distance between genotypes, mean diameter at breast height (DBH), and number of ramets on male reproductive success, ii) the influence of maternal clone genotype, year, female phenology, and DBH on female mate choice, and iii) the average genetic correlation among offspring for each open-pollinated family, each year represented in the progeny tests, and in the overall offspring population.

## Materials and methods

A northern red oak seed orchard at the Indiana State Tree Nursery in Vallonia, Indiana (N 38.847, W 86.098) served as the study population. The Vallonia seed orchard (VSO) was established using grafts from plus-trees selected throughout the native range of northern red oak. The grafts (ramets) are genetically identical to the individual tree (ortet) from which they came. Because the ramets are copies of genotypes from many provenances and stands throughout eastern North America, the seed orchard is expected to be a heterogeneous, unstructured population. The VSO consists of five sublines, or smaller orchards, separated by 150 to 1700 meters (S1 Fig). The seed orchard, planted between 1986 and 1988, was designed so that each subline would act as an individual breeding unit. At the time of establishment, each subline contained 30 clones randomly assigned to a subline. Each subline contains a unique set of clones. There were 8 ramets per clone for a total of 240 trees per subline, or 1200 total trees in the orchard. Initial spacing was 8 x 8 m. High mortality occurred due to delayed graft incompatibility, so at the time the research took place each subline contained 19 to 27 clones with 1 to 7 ramets per clone (mean 4.6±1.7).

### Phenology groups

Phenology data was collected in the spring of 2008, 2009, and 2010 [21]. Trees were observed beginning on or near April 1 each year and phenology data collection began when the earliest individual(s) reached bud break stage. Phenology data was collected every 2 to 3 days in 2008 and 2009 and every 4 days in 2010. Male flowers were scored from the ground using binoculars and female flowers were assessed by bending down branches, climbing trees, or from an aerial lift. Data collection continued until the phenologically latest individual(s) completed pollen shed.

All variables were assessed in the top 50% of the tree crowns and refer to the onset of the physiological process being described. Bud break was recorded for an individual when the scales covering the bud were visibly opened, just before the new shoot began growth. Onset of pollen shed was recorded when observed catkins had at least one pollen sac opened with extended anthers, peak pollen shed when observed catkins had 75–100% of pollen sacs open, and the end of pollen shed was determined when catkins were dark brown, dry, and no longer dehiscing pollen. Leaf out was recorded at the appearance of the first fully expanded green leaves. Clones were grouped into early, middle, and late phenological classes based on the average timing of beginning pollen shed of their ramets. Early (E) clones began pollen shed within seven days of the earliest clone, late (L) clones ended pollen shed within seven days of the latest clone, and all other clones were classified as intermediate (M). Clones with no flowering ramets were placed in a phenological class based on leaf out date [21]. Wind speeds in central Indiana average 18.0 kph in April and 15.5 kph in May.

### Microsatellite genotyping

A leaf sample was collected from each of 349 individuals trees in the VSO in June 2008, and DNA was extracted from fresh leaves by mechanical grinding in a modified CTAB buffer [22] followed by two rounds of chloroform/isoamyl extraction or one plant tissue cycle in an AutoGen™ (AutoGen Inc., Holliston, MA, USA) automated nucleic acid extractor according to manufacturer’s instructions. Ten clones were chosen from the VSO that represented the early, intermediate, and late phenology groups [21] and had offspring in progeny tests throughout Indiana. Surviving offspring of the selected 10 clones were sampled in July 2009 and DNA was extracted as above, except DNA was extracted from fresh or lyophilized leaves. A total of 756 offspring of the 10 selected clones were planted in 2007 and 2008; 601 were living at the time of collection. DNA was extracted from a total of 950 northern red oak trees.

After removal of 61 individuals with low DNA quality, 889 individuals were genotyped with 11(GA)_n_ microsatellite loci previously tested in northern red oak [23,24]. PCR-based genotyping was carried out with the QIAGEN Multiplex Kit (catalog No. 206143) with PCR conditions following manufacturer’s recommendations: 1X Multiplex master mix (final concentration of 3mM MgCl_2_), 0.2uM each forward-labeled primer, approximately 20 ng genomic DNA and addition of water to a final reaction volume of 12.5 uL. Gradient PCR was carried out on all multiplexes, and the final thermal profile was: 5 minutes of denaturation at 95°C, followed by 28 cycles of denaturation at 95C for 30s, annealing at 54–59 for 90s, and extension at 72 for 30s, with a final extension at 60 for 30min. Fragment sizes of the amplified labeled microsatellites were determined using an ABI-PRISM 310 genetic analyzer (Applied Biosystems, Foster City, CA, USA). Alleles were confirmed visually using GeneMapper® software version 3.7 (Applied Biosystems, Foster City, CA, USA). Individuals of known genotype and an artificial sample of the seed orchard’s ‘reference alleles’ (a product of amplification of DNA mixed from several parental genotypes representing the parental population’s allele range) were included to ensure consistent scoring.

### Data analysis

Data analysis was based on a total of 781 individuals after excluding any individual with four or more loci drop-outs caused by poor amplification or ambiguous size scoring. The multi-locus consensus genotype was determined for each VSO clone (a total of 146 parental genotypes representing the 349 orchard ramets) and 432 offspring. Identity tests, parent-pair analyses, and paternity tests were performed with the software CERVUS 3.0.3 [25,26] with relaxed (80%) and strict (95%) assignment probability and allowance for genotyping error. Offspring with either a strict (95%) paternal assignment or a non-zero parent-pair delta value were used for further statistical analysis. Regressions, chi-square analyses, and ANOVAs were generated using SAS/STAT® software, Version 9.1 of the SAS System for Windows (SAS Institute Inc., Cary, NC, USA).

Variables assessed to determine influences on female mate choice were individual genotype, year, female phenology class, and mean diameter at breast height (DBH). Variables assessed to determine influences on overall male reproductive success and male reproductive success per female were male subline location, male phenological class, male subline, distance between mother and candidate male, mean DBH, and number of ramets per clone. Mean DBH was determined for each clone on the basis of ramets remaining as of April 20, 2010. Distances between mother tree and candidate males were categorized based on the distance between the subline of the mother tree and the subline of the candidate male. Straight-line distances were not used because offspring were bulked by maternal genotype; thus, the ramet from which the offspring came is unknown. Within-subline pollinations were given a value of 1, pollinations from neighboring sublines were given a value of 2, pollinations from non-neighboring sublines in the main orchard area were given a 3, and all pollinations from males in subline D (approximately 1 km outside the main orchard area) were given a distance value of 4.

Male reproductive success was determined by counting the total number of offspring each male contributed to the overall offspring pool (n = 336) and by counting the number of offspring each male contributed to the total offspring of each mother tree (n = 22 to 46). Mean mating frequency for each clone was calculated as the ratio of a single clone’s sired offspring to the total number of offspring. Total count data was transformed in SAS to relieve unequal error variance according the best transformation (λ = -2) indicated by the Boxcox option of the SAS TRANSREG procedure. Male reproductive success expressed as proportions of total offspring or proportions of offspring attributable to each mother tree were inverse transformed (λ = -1) in the same manner. Model statistics are presented for models using transformed data; least squares means and standard errors are presented untransformed.

### Average genetic correlations

There are four possible genetic correlation values for offspring *i* and *j* (*GC*_ij_) in an OP family when the parents are non-inbred and non-correlated: self full-sibs (*GC*_ij_ = 0.667), full-sibs (*GC*_ij_ = 0.500), self half-sibs (*GC*_ij_ = 0.408), and half-sibs (*GC*_ij_ = 0.250). Knowing the proportion of each type of relationship allows the calculation of *GC*_ij_ for the *n*th family as *GC*_ij_(*n*) = *GC*_sfs_ × *p*_sfs_ + *GC*_fs_ × *p*_fs_+ *GC*_shs_ × *p*_shs_+ *GC*_hs_ × *p*_hs_ where p is the proportion of the respective type of relative [19]. Based on the results of the paternity analysis, the proportions of the various relatives as well as the average genetic correlations were calculated for each OP family, each year represented in the progeny tests, and in the overall offspring population.

## Results

### Paternity analysis

A total of 349 northern red oak seed orchard parent trees and 432 offspring were genotyped at 11 microsatellite loci for paternity analysis. The 349 parent trees were grafted clones representing 146 unique genotypes. Paternity analysis performed most efficiently with the nine microsatellite loci described in Table 1. Loci were highly polymorphic and ranged between 12 and 52 alleles per locus with a mean of 31 alleles per locus. Observed heterozygosity ranged between 0.72 and 0.92 with a mean of 0.83. Minimum polymorphic information content (PIC) was 0.77 and maximum PIC was 0.95 with a mean of 0.88 over all loci (Table 1). Mean proportion of individuals typed over all loci was 0.89, and combined non-exclusion probabilities for one parent, one parent given the other parent, parent-pairs, and identity tests were 0.00003479, 0.00000037, 6.41E^-12^, and 1.09E^-16^, respectively.

**Table 1:** Diversity statistics of microsatellite loci used to type northern red oak seed orchard parents and offspring.

Locus	A*	N	*H*_O_	*H*_E_	PIC
*quru10*	19	671	0.876	0.888	0.878
*quru4*	36	669	0.918	0.949	0.946
*quru9*	38	653	0.778	0.818	0.801
*quru12*	12	599	0.721	0.797	0.772
*quru5*	52	590	0.842	0.926	0.92
*quru8*	30	619	0.855	0.944	0.941
*quru11*	39	574	0.742	0.933	0.928
*quru3*	34	554	0.827	0.888	0.878
*quru2*	19	660	0.894	0.876	0.864

*A, number of alleles; N, number of individuals typed; *H*_O_, observed heterozygosity; *H*_E_, expected heterozygosity; PIC, polymorphic information content. N = 697 trees. Parents are located in a clonal seed orchard; offspring are located in three progeny tests established in 2007 and 2008 in Indiana, USA.

An identity test was performed to check for misidentified clones and trees in the orchard. In total, 9 of 146 genotypes had at least one ramet mislabeled with a mean probability of identity (pID) of 1.22E^-11^. Eleven ramets of 349 were mislabeled. Eight ramets were mislabeled and belonged to a different genotype within the orchard. Three ramets were relabeled after they were found to be identical to another orchard genotype.

Paternity was analyzed using a strict (95%) confidence level and a relaxed (80%) confidence level for 432 offspring and 143 paternal genotypes. When paternity was analyzed given a known mother (i.e., each seed orchard clone except the known female was considered a candidate male), 61% of progeny were assigned a male parent within the orchard at the 95% confidence level and 93% were assigned paternity at the 80% confidence level. Seven percent of progeny were not assigned a male parent within the orchard. Mean observed genotyping error rate across all loci was 0.057. Parent pair analysis was also conducted using Cervus 3.0.3 and previously mentioned simulation parameters. Offspring had an average proportion of 0.973 sampled parents and an average of 10585 candidate parent pairs. Sixty-two percent and 91% of offspring were assigned a parent-pair within the orchard at the strict and relaxed confidence levels, respectively. Nine percent of offspring were not assigned a parent-pair within the orchard. A total of 336 offspring had either strict (95%) paternal assignment or a non-zero parent-pair delta value and were retained for analysis of pollen gene flow, female sampling, male reproductive success, and genetic correlations.

### Pollen contamination

Clones were ranked as phenologically early, intermediate, or late by the timing of onset of male flowering or onset of leaf out over three years [21], and there was a significant association between percent paternity assignment and maternal phenology group (χ^2^ = 17.83, *p* = 0.0001). Included in the analysis were 178 offspring from maternal parents in the early phenology group, 144 offspring from maternal parents in the intermediate phenology group, and 110 offspring from maternal parents in the late phenology group. Of the 178 offspring from maternal parents in the early phenology group, 154 (87%) were assigned to fathers within the seed orchard, 137 of the 144 (95%) offspring of intermediate phenology maternal parents, and 109 of 110 (99%) offspring with late phenology maternal parents were assigned paternity within the orchard.

### Female mating success

The ten grafted clones of northern red oak used in the mate choice study had OP offspring in progeny tests from two different years’ pollinations. Chosen clones represented each of the phenological groups and all four accessible VSO sublines. Five clones served as mothers for 110 offspring in 2007 and ten clones (inclusive of the previous year’s five) served as mothers for 226 offspring in 2008. A mean of 33.6 offspring per clone was sired by a mean of 19.9 fathers, for an overall mean of 1.69 offspring per male on a given female tree. A two-way ANOVA general linear model containing clone and year explained a significant amount of the variance in female sampling rate (*R*^2^ = 0.960, *p* = 0.0215, Table 2). Clones varied in sampling rate from a mean of 1.36 offspring per male to a mean of 2.83 offspring per male, and varied significantly in their mean sampling rates (Table 3). In 2007 and 2008, the mean number of offspring per male, 1.26 and 2.11, respectively, was also significantly different (*p* = 0.006). Phenological class did not explain a significant amount of variation in number of offspring per male (*F* = 0.73, *p* = 0.504). Maternal trees in the late phenological group had the highest number of offspring per male, followed by the intermediate group and the early group, although none of the phenology groups were significantly different for this trait (Table 3).

**Table 2:** Analysis of variance in female sampling rate measured as number of male parents in the offspring pool of ten grafted northern red oak seed orchard clones in Indiana, USA.

Source	DF	SS	MS	*F*	*p* > *F*
Model	10	4.23	0.423	9.61	0.0215
Error	4	0.176	0.0441		
Corrected Total	14	4.41			
Clone*	9	2.97	0.330	7.48	0.0341
Year^**^	1	1.26	1.26	28.7	0.0058

*‘Clone’ is the maternal genotype.

**Year of progeny test establishment.

Type I SS shown for individual sources of variation; α = 0.05.

**Table 3:** Least squares means for sources of variation in female sampling rate in a northern red oak seed orchard clonal seed orchard in Indiana, USA.

Source of variation	Sampling rate mean	Standard error	Tukey group
Clone*			
70	2.48	0.220	a^‡^
65	2.20	0.220	ab
41	1.92	0.220	abc
33	1.90	0.149	abc
132	1.89	0.220	abc
165	1.77	0.149	bc
112	1.52	0.149	bc
6	1.46	0.149	cd
107	1.40	0.149	cd
123	1.01	0.220	d
Year**			
2007	1.40	0.115	a
2008	2.11	0.0664	b
*Phenological group*^*†*^			
Early	1.62	0.256	a
Intermediate	1.80	0.256	a
Late	2.06	0.256	a

Female sampling rate measured as the number of male parents represented in the offspring pool of each clone.

*‘Clone’ is the maternal genotype.

**Year of progeny test establishment.

^†^Phenological group of the maternal genotype.

^‡^ Class level lsmeans in different Tukey groups are significantly different at the α = 0.05 level.

Maternal trees from all three phenology groups were most often pollinated by males in the intermediate phenology group. Thirty-four percent of early offspring, 39% of intermediate offspring, and 56% of late offspring were pollinated by males in the intermediate phenology group, respectively (Table 4). All mother trees had offspring sired from males in all three phenology groups. A chi-square test for association revealed no significant association between phenology group of the female parent and phenology group of the male parent (*χ*^2^ = 5.279, *p* = 0.2608). There was not a significant correlation between size of the maternal half-sib family and number of sires.

**Table 4:** Number and percent of northern red oak progeny test offspring from grafted seed orchard maternal clones in early (E), intermediate (M), late (L), and unknown (u) phenological classes by paternal phenological class.

Maternal phenological class	Paternal phenological class				
Early	E	I	L	u	Total
Number	23	35	21	23	102
Per cent	23%	34%	21%	23%	100
Intermediate	E	I	L	u	Total
Number	27	38	21	12	98
Per cent	28%	39%	21%	12%	100
Late	E	I	L	u	Total
Number	18	51	16	6	91
Per cent	20%	56%	18%	7%	100

### Male reproductive success

**Fig 1:**
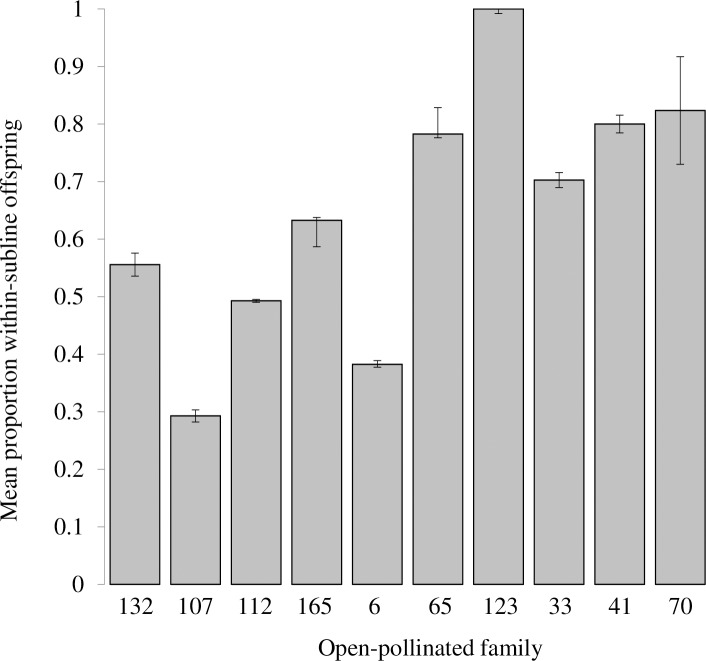
Ranked frequency distribution and null-expectation (broken line) male reproductive success in a clonal northern red oak seed orchard in Indiana, USA. Reproductive success for years 2007 (left, 1.69 expected) and 2008 (right, 3.05 expected) based on paternity analysis of 336 progeny test offspring.

**Fig 2:**
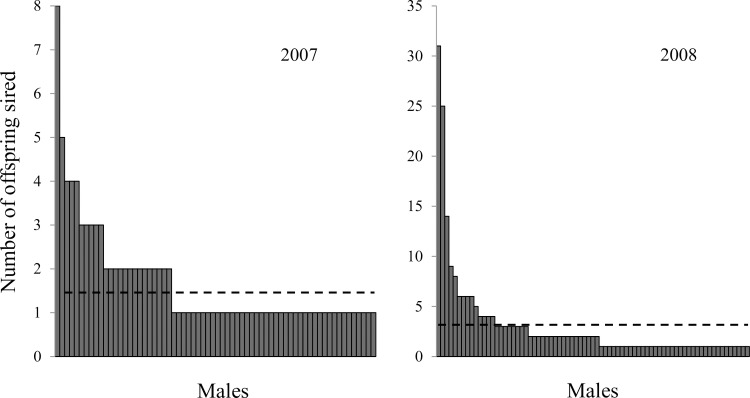
Regression of number of offspring sired on number of female mates for 99 northern red oak clones serving as male parents in a grafted northern red oak seed orchard in Indiana, USA. Saturation of symbols denotes the number of observations for that coordinate pair: light gray = 1 to 5; dark gray = 6 to 19; black = 20+.

At the time of pollination (spring 2005 and 2006), there were 146 northern red oak clones in the VSO. The mean number of male parents per year was 69.5, or 47.6% of all clones. Ninety-nine clones in the seed orchard, or 67.8%, served as male parents in at least one of the two pollination seasons from which the progeny tests were established. The overall mean mating frequency was 0.011, with a minimum mating frequency of 0.003 and a maximum of 0.099. In the 2007 offspring, 75% of fathers sired only one offspring. There were 110 offspring from the 2007 cohort assigned high-quality paternity within the orchard to 65 males. In the 2008 offspring, 60% of fathers sired one offspring, and there was a total of 226 high-quality offspring sired by 74 males. In 2007 and 2008 the maximum number of offspring sired by a single male was 8 and 31, respectively. Most males sired only one to three offspring, with a few males siring a relatively large number of offspring (Fig 1). Some clones sired significantly higher overall number of offspring than others. Four of 99 clones sired a disproportionate number of total offspring, and one clone, 53, sired significantly more offspring than the other clones. Clones that sired relatively more offspring than other clones were sampled by more females (*R*^2^ = 0.555, *p*<0.001, Fig 2). An exponential regression formula (y = 0.7654e^0.6129x^) best fit the relationship of male mating frequency (independent variable) and female sampling (dependent variable).

**Fig 3:**
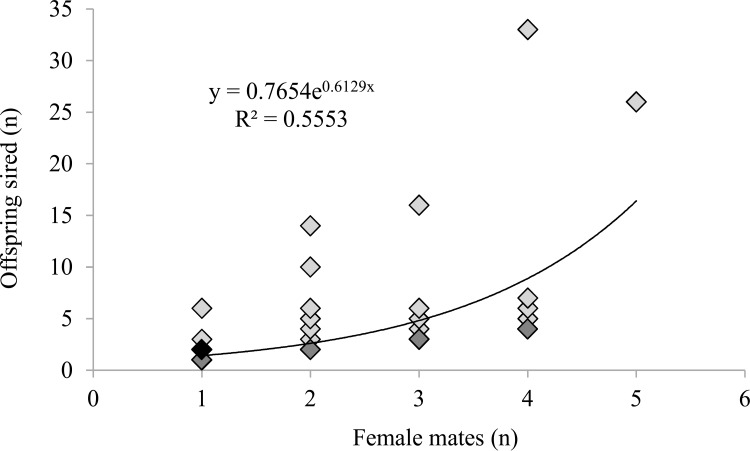
Mean proportions of within-subline matings for ten maternal genotypes in a clonal northern red oak seed orchard in Indiana, USA. Error bars are the standard error of the mean proportion across males for each maternal genotype (average number of males per maternal genotype: 19.9±4.04; range: 6 to 47 males. Families are ordered from left to right by maternal phenological rank. Average size of OP family: 33.6±5.06; range: 15 to 67 offspring. Total offspring pool was 336 open-pollinated progeny.

In an analysis of variance general linear model containing male subline, male phenology group, mean DBH, and number of ramets per clone, only male subline explained a significant amount of the variation in number of offspring in the overall progeny pool (*F* = 3.36, *p* = 0.0236). Pollination occurred most often by male trees in the same subline as the maternal tree regardless of phenological class. There was a significant association between the subline of the female parent and the subline of the male parent (χ^2^ = 246.2, *p*<0.0001). The highest proportion of offspring from each mother tree was sired by males in the same subline (Fig 3). There was a significant association between phenological class and number of within-subline matings, (χ^2^ = 8.99, *p* = 0.019), with phenologically late clones having a significantly higher proportion of within-subline matings than early clones or the average of early and intermediate clones, Table 5).

**Table 5:** Proportion of northern red oak progeny test offspring from ten maternal clones in the early, intermediate, or late phenological class resulting from within-subline pollination.

Maternal Phenological class	Proportion within-subline mating
Early	0.48 ± 0.083*a*
Early + Intermediate	0.52 ± 0.072*a*
Intermediate	0.60 ± 0.12*ab*
Late	0.83 ± 0.062*b*

Proportions of within-subline matings followed by different letters were significantly different at the α = 0.05 confidence level.

Male parents in the early phenology group (n = 13) sired higher numbers of offspring (5.31±1.44) than male parents in the intermediate phenological group (n = 38) or the late phenological group (n = 19; mean number off offspring per male 3.71±0.55 and 3.37±1.28, respectively).

There were 185 parent-offspring pairs in the 336 high-quality paternity calls, ranging between 1 and 31 offspring per pair. For each mother tree, variance in number of offspring per male was best described by a model containing geographic distance and male phenology (*R*^2^ = 0.207; *F* = 7.44, *p* < 0.0001). The average number of offspring from mother trees by males in the distance category “1” (within-subline) was 2.75±0.24, significantly higher than any other distance category. Males in distance categories 2, 3, and 4 sired an average of 1.49, 1.24, and 1.37 offspring, and means were not significantly different. Males in the early phenology group sired an average of 2.40±0.41 offspring per mother tree, significantly more than males in the intermediate (1.46±0.27) or late (1.52±0.35) phenological groups (Table 6).

**Table 6:** Least squared means for significant sources of variation in male reproductive success in a five-subline grafted northern red oak seed orchard in Indiana, USA.

Source of variation	Number progeny lsmean	Standard error	Tukey group
Distance*			
1	2.75	0.24	a**
2	1.49	0.28	b
3	1.24	0.35	b
4	1.37	0.51	b
Paternal phenology			
Early	2.40	0.41	a
Late	1.52	0.35	ab
Intermediate	1.46	0.27	b

Offspring from ten maternal genotypes were sampled. Male reproductive success estimated as the number of progeny test offspring per seed orchard female.

*Distance category ‘1’ represents within-subline pollinations, ‘2’ neighboring sublines, ‘3’ non-neighboring sublines in the main orchard area, and ‘4’ represents pollinations from a subline approximately 1 km south of the main orchard area.

**Class level lsmeans in different Tukey groups are significantly different at the α = 0.05 level).

### Average genetic correlations

**Fig 4:**
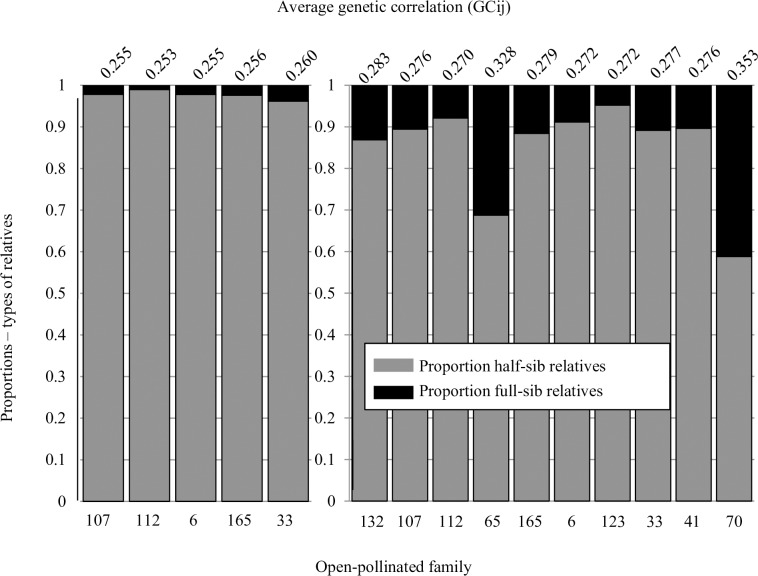
Proportions of half-sibs and full-sibs in five open-pollinated (OP) families established from a clonal northern red oak seed orchard in 2007 (left) and 2008 (right). Average genetic correlation between offspring pair *ij* (*GC*_ij_) was calculated for each family and is presented above each bar. Families are ordered from left to right by phenological rank. Average size of 2007 OP family: 18.8±1.46; range: 15 to 23 offspring; average size of 2008 OP family: 20.9±1.36; range: 15 to 27 offspring.

The average genetic correlation (i.e., genetic relatedness) among offspring and the proportion of full and half-sib pairs from each mother tree (based on 336 high-quality paternity calls) were used to determine the average genetic correlation for each OP family, year, and the total offspring pool. Proportions of half-sibling pairs in the overall offspring pool for each OP family ranged between 0.022 and 0.413, and the proportion of half-sibling pairs (0.0232±0.0045) in the 2007 cohort was significantly lower than the proportion of half-sibling pairs (0.150±0.037) in the 2008 cohort (*t* = -2.39, *p* = 0.016). The average genetic correlation among OP progeny in the 2007 cohort ranged between 0.253 and 0.260, with a mean of 0.255±0.001. The average genetic correlation among OP progeny in the 2008 cohort ranged between 0.270 and 0.353 with a mean of 0.289±0.009 (Fig 4).

Average genetic relatedness among all OP progeny ranged between 0.256 and 0.352 with a mean of 0.281±0.01 (Table 7). For mother trees having offspring in both the 2007 and 2008 cohorts, the ratio of overall *GC*_ij_ to mean yearly *GC*_ij_ was calculated by dividing the mean *GC*_ij_ over 2007 and 2008 by the overall *GC*_ij_ (found by pooling offspring across cohorts). A ratio of < 1 represents an overall correlation that is lower than the yearly average (i.e. there are more full-sib pairs in a given year than in the total pool), while a ratio of >1 represents on overall correlation that is higher than the yearly average. Of the five families with two cohorts, all but one (clone 33) had a ratio of overall *GC*_ij_ to mean yearly *GC*_ij_ that was < 1. Similarly, the average *GC*_ij_ of OP families each year was higher than the overall genetic correlation for that year (Table 7).

**Table 7:** Average genetic correlation (*GC*_ij_) among offspring of 10 northern red oak open-pollinated families from a clonal seed orchard in Indiana, USA.

Family	Overall genetic correlation (*GC*_ij_) among offspring	Ratio of overall to mean yearly (*GC*_ij_)*
112	0.2557	0.979
6	0.2602	0.986
107	0.2610	0.982
123	0.2619	
165	0.2620	0.980
33	0.2706	1.009
41	0.2758	
132	0.2828	
65	0.3281	
70	0.3529	
Mean	0.2811	
Year		
2007	0.2530	0.989
2008	0.2601	0.904
Mean	0.2566	

*The ratio of overall *GC*_ij_ to mean yearly *GC*_ij_ is presented for each family with offspring in both the 2007 and 2008 progeny tests. Mean yearly *GC*_ij_ for families are averaged over two years and mean yearly *GC*_ij_ for years are averaged over families (5 in 2007, 10 in 2008).

## Discussion

### Paternity analysis

Assignment of paternity is most successful in OP mating designs when the maternal genotypes are known, the size of the candidate parent pool is small, genotyping errors are accommodated, and the information content of the genetic makers is high [25,27]. First-generation seed orchards are especially suited to paternity assignment as parent clones are usually unrelated; as breeding programs advance, paternity assignment loses power due to increased coancestry among individuals [27]. With a mean of 31 alleles per locus, the microsatellites we used provided a combined non-exclusion probability of 3.7E^-7^, or a 1 in 37 million chance of failing to exclude an unrelated male. In this study, high non-exclusion probability combined with known maternal genotypes and the relative isolation of VSO from natural stands containing *Quercus* led to 93% of offspring having paternity assigned within the seed orchard, with 61% of those assignments at the 95% confidence level.

### Pollen contamination

Studies of gene flow and mating success in hardwood species have taken place largely in natural stands, and a common trend among oak studies is a high percentage of offspring with male parents outside the study sites. In this study, extra-orchard pollen accounted for 7% of all offspring, an extremely low number compared to reports from pine seed orchards, where between 14 and 74% contamination was reported [28], and a *Quercus robur* seed orchard where 70% of offspring was assigned paternity outside the orchard [10]. Two studies in a *Eucalyptus grandis* seed orchard found pollen contamination rates of 29% [29] and 39% [10]. The prevalence of agricultural land surrounding the VSO and a large suite of markers with high exclusion probability likely aided in the high assignment rate.

### Pollen gene flow

Within the orchard, pollen gene flow between sublines varied from 30 to 60%. In a sublining system, this would not serve to decrease genetic gain (as all sublines are at the same stage of selection), but it would increase coancestry in further populations selected from the VSO where offspring from mother trees in different sublines are assumed to be unrelated.

Offspring from phenologically early mother clones had a higher pollen contamination rate (13%) than intermediate (5%) or late (<1%) offspring. VSO data indicates that early blooming trees bloom longer, such that female flowers of phenologically early trees may be exposed to pollen over a longer period of time, increasing the chances of contacting extra-orchard pollen. Bai *et al*. [30] suggested that very early and/or late individuals in phenologically variable populations of blue oak (*Quercus douglasii*) may have reduced reproductive success that contributes to reduced fertilization in the population as a whole. Thus, when the northern red oak pollen cloud density is low during the earliest and latest periods of flower production in the orchard, near neighbors may become relatively more important as pollen parents.

### Female mating success

In this study, 96% of the variance in sampling rate was explained in an ANOVA model containing year and maternal genotype. Between-year variation was the more strongly significant, reflecting the influence of local environmental factors during pollination season. Regression analysis indicated males that produced relatively many offspring mated preferentially with a limited number of females rather than mating evenly across females (Fig 3). As this could be an indication that specific combining ability influenced nursery and early plantation survival, these results should be compared with similar studies using seed crops rather than seedling offspring.

In this study, female phenology group did not explain a significant amount of variation in mating success. Intermediate males sired most offspring, which is not surprising because most trees fell into this category. Taken together, the lack of variation in female sampling rate (i.e., the number of males used by each female) irrespective of phenology and the lack of association between the phenology group of successful sire and dam combinations, along with the successful mating of males from all phenology groups on females of all phenology groups, underscores the insignificance of phenological differences on sampling of pollen by female flowers.

### Male reproductive success

Differential male reproductive success has been reported in several conifer species, presumably due to phenological mismatch, variation in male fertility, or postzygotic barriers such as embryo abortion [3,31,32]. In OP systems, however, many factors can contribute to stand level fertility variation that is not present in controlled settings. Floral asynchrony, floral abundance, tree location and size, and management techniques can all contribute to unequal male contributions in OP seed orchards. Unequal male reproductive success in OP seed orchards has been reported for Norway spruce (*P*. *abies*, [32]), white spruce (*Picea glauca*, [33]), and red spruce (*P*. *rubens*, [34]). In an *Abies alba* seed orchard with 12 clones, Hansen [35] determined that 80% of the progeny was sired by four clones. Similarly, two studies of *A*. *nordmanniana* revealed that 3 of 13 clones sired 75% of seeds [36] while five clones out of 23 sired 60% of 570 progeny test offspring in a separate test [37]. Three of six *Eucalyptus urophylla* clones in a production seed orchard sired 148 of 149 sampled offspring in a single-year progeny test [29], and 199 of 349 (57%) potential male trees sired 440 offspring at variable rates in *E*. *grandis* [20].

In the present study about half of the orchard clones served as male parents each year, and 99 of 146 (68%) clones were represented as males in the total offspring pool (n = 336) with each sire contributing between 1 and 31 offspring. Flowering data from 2008 to 2010 showed that between 83 and 101 clones (86 to 97%) had male flowers each year [21]; thus, there were likely flowering clones that did not sire any offspring during the 2005 and 2006 pollination seasons. Seventy percent of the 2007 (n = 110) and 2008 (n = 226) progeny was sired by 28 and 25 males, respectively. Four males sired almost 30% of the total progeny pool, with the top two most successful siring 18% of sampled progeny (Fig 2).

Trees were most often pollinated by clones in their own subline. Males in increasingly distant sublines did not have increasingly lower mating success; rather, males in distance categories 2, 3, and 4 sired an average of 1.49, 1.24, and 1.37 offspring per maternal clone, respectively. Despite reports of common long-distance gene flow, these data support the important role of short distance gene flow. Average pollination distance in *Quercus lobata* was estimated to be 65 m [38], and acorn production in *Q*. *douglasii* was positively correlated with the number of flowering trees within 60–80 m [39]. Like Kimura *et al*. [16], our data indicated increased male reproductive success at relatively short distances, as VSO sublines are approximately 60 m by 40 m in area and separated by at least 200 m.

Male subline (i.e, distance from the maternal tree) and male phenology group explained a significant amount of variation in male reproductive success. Number of ramets and ramet size, estimated by DBH, had no influence on male reproductive success. Strobili abundance has been shown to influence male reproductive success in *Picea* [37], but we could not estimate catkin abundance in most of the 5–25 m tall crowns.

Males in the early phenology group sired an average of 2.40 ± 0.41 offspring per mother tree, significantly more than males in the intermediate (1.46 ± 0.27) or late (1.52 ± 0.35) phenological groups (Table 6). Thus, phenologically early males may not contact as many female flowers as intermediate males, but early males sire proportionately more offspring with females they do contact. This may be attributed to low pollen cloud density and lack of stigmatic competition at an early flowering date. Late males did not have similar success, even though slight protandry ensured the presence of female flowers for late males. The enhanced reproductive success of early males was likely due to the longer duration over which they flowered. The length of time a ramet will produce flowers is largely determined by the extent of favorable environmental conditions; as weather becomes warmer or rain events interrupt the flowering period, those ramets that flowered first will have flowered longest [21]. The duration hypothesis is further supported by higher pollen contamination rates observed among offspring from early clones that have fewer nearby flowering males than later clones. Thus, distance and phenology may have had distinct roles in influencing male reproductive success, with distance largely dictating how many male and female genotypes were available to one another, and phenological class determining how much time they have to interact. Early female flowers had the longest time to sample any available male pollen, and early male flowers had time to increase reproductive success with any available females.

### Average genetic correlations

The skew in mating success indicated by studies in conifers and hardwoods indicates that the progeny of a single parent rarely constitute a true half-sib family. In theory, the average genetic correlation among OP offspring could range from 0.25 (all half-sibs) to 0.5 (all full-sibs). For the purposes of calculation of quantitative genetic parameters, what is desired is not random mating among all ramets but a consistent level of genetic correlation among offspring that matches expectations (0.25 for seeds from open-pollination from each female parent).

Conifer seed orchard managers have proposed using intermediate values between 0.30 and 0.33; however, those values were based on anecdotally observed levels of self-pollination and phenological differentiation, and lacked data-based support [40,41]. Authors studying *Pinus* and *Picea* have also suggested the seed crop from their orchards had a *GC*_ij_ higher than 0.25 due to the occurrence of self-pollinated progeny, non-equal pollen contributions, and unaccounted relatedness between parents [3,40,42,43]. However, Gaspar *et al*. [44] directly estimated a mean *GC*_ij_ of 0.26, or an average of about 4% full siblings, using a suite of microsatellite markers on five *Pinus pinaster* families (25 offspring per family). In an *Abies nordmanniana* clonal seed orchard, Hansen and Nielsen [37] found an average genetic correlation of 0.29 within OP families (range of 0.26–0.33). Similarly, Kumar and Richardson [45] found an average genetic correlation of 0.29 among *Pinus radiata* offspring. Genetic correlation values for the 10 northern red oak families in the present study ranged between 0.256 and 0.353, with a mean of 0.281. Three of the 10 families had genetic correlation estimates above 0.28; two had estimates greater than 0.30. This high incidence of full-sib relationships and the resulting among-family variability in *GC* values are conditions that may significantly affect heritability estimates; indeed *GC* values of 0.28 are enough to produce heritability overestimates of 10% or greater [44].

Offspring in the 2007 and 2008 cohorts had an average *GC*_ij_ of 0.253 and 0.260, respectively. Similarly, offspring in a *Quercus robur* stand showed relatedness between 0.26 and 0.27 after unrelated individuals were identified and removed [46]. Heritability for *P*. *pinaster* tree diameter was slightly higher though not significantly different when calculated with an assumed *GC*_ij_ of 0.25 (*h*^2^ = 0.166) than with the estimated *GC*_ij_ of 0.26 (*h*^2^ = 0.155, [44]). Heritability for five of six characters in *A*. *nordmanniana* did not improve when paternity information was included in the calculation, however dominance effects and skewed male reproductive success caused the heritability of height to be badly overestimated (*h*^2^ = 0.57±19 unadjusted, 0.35±12 adjusted; [37]. Thus, heritability estimates based on the yearly *GC* values 0.25 and 0.26 found in this study would be robust for most traits, given unbiased estimates of average relationship among offspring within families [44].

### Practical considerations

Based on these calculations and empirical data from conifer seed orchards, it can be concluded that in *Quercus rubra* open-pollinated progeny tests, the error in heritability estimates due to the inclusion of full-siblings is low, and heritability and other genetic parameters may reasonably be estimated from one or more OP offspring cohorts without the inclusion of progeny genotype data. Concerning the importance of distance to orchard panmixia versus the stated goal of producing seedlings in sublines, it is clear that sublines in this system are not isolated. Trees not in the same subline orchard will have a similar, low but consistent rate of mating, irrespective of distance.

Early blooming males were more successful than males in the intermediate (most common) or late phenology classes. On the practical level, this means that orchard managers can predict which parents are likely to be overrepresented in the seedlot based on easily observable phenological traits. It will be useful to learn what selection forces stabilize phenology in wild populations, counteracting the apparent advantage of trees with early phenology (perhaps frost, which was not a factor in the years of our study). Data from our study shows that trees with late male bloom fertilize early blooming females. Orchard managers cannot assume that phenology determines which trees are mating, and, conversely, they can assume that if they add a new selection to the orchard, even if it is late blooming, it has a good chance of crossing with all other females, even early ones.

Our data showed that among the progeny of a typical year, nearly all parent trees are represented at least once, and relatively few are greatly overrepresented. This meets the expectation of landowners that seedlings they buy from the nursery will be genetically diverse. Finally, progeny test genotyping also uncovered clones that acted as sires only; thus, breeding values can be estimated for clones that made no seeds when microsatellite-based paternity analysis is employed, whereas no such information would be available in a traditional OP breeding program.

## Supporting information

S1 Fig Layout of the Vallonia Seed Orchard. Top: Sublines A, B, C, and E of the Northern Red Oak Seed Orchard in Vallonia, Indiana. Subline D (not shown) is approximately 1 km south of Subline C. Bottom: Subline A overview (left) and detail (right).
(DOCX)
