# Supplementary material for: Pollen gene flow, male reproductive success, and genetic correlations among offspring in a northern red oak (Quercus rubra L.) seed orchard
Source: PLoS One. 2017 Feb 6;12(2):e0171598. doi: 10.1371/journal.pone.0171598 (PMC5293549; doi:10.1371/journal.pone.0171598)
Supplement: S1 Fig — Top: Sublines A, B, C, and E of the Northern Red Oak Seed Orchard in Vallonia, Indiana. Subline D (not shown) is approximately 1 km south of Subline C. Bottom: Subline A overview (left) and detail (right). (DOCX) [file pone.0171598.s001.docx]

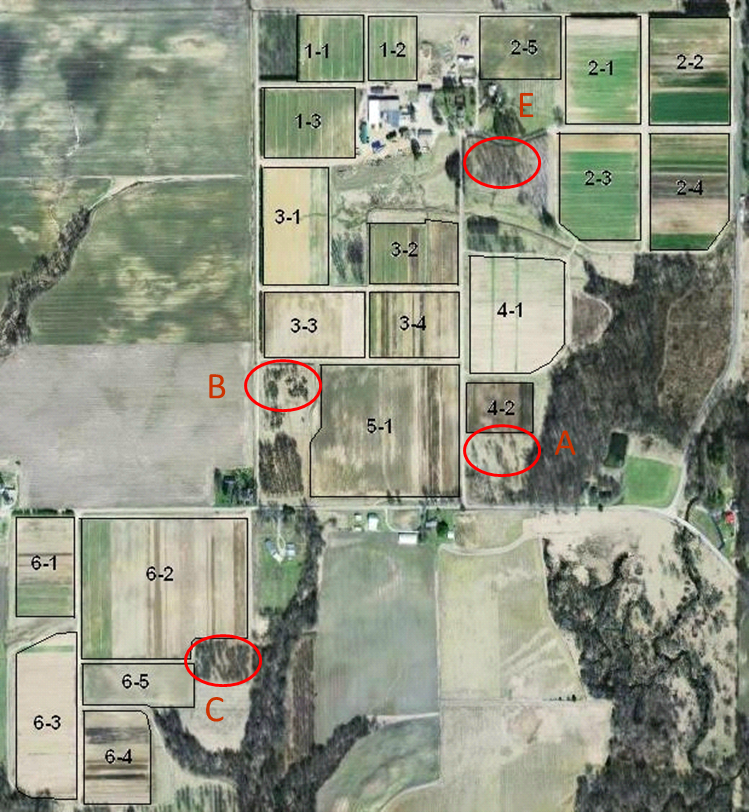
**Fig S1. Layout of the Vallonia Seed Orchard.**

**50 m**

Top: Sublines A, B, C, and E of the Northern Red Oak Seed Orchard in Vallonia, Indiana. Subline D (not shown) is approximately 1 km south of Subline C. Bottom: Subline A overview (left) and detail (right).
